# The Book World of Medicine and Science

**Published:** 1902-01-18

**Authors:** 


### The Book World of Medicine and Science.

The Journal of Obstetrics and Gynæcology of the British Empire. Vol. I. No. 1. (London: Baillière, Tindall & Cox. January 1902. Price 2s. 6d. net.)

This new journal starts its career with a fine show of talent on its cover and a very good series of articles within. It is edited by Alban H. G. Doran, F.R.C.S., of the Samaritan Hospital, with the aid in special departments of D. Berry Hart, M.D., Lecturer in Midwifery and Diseases of Women, Royal College, Edinburgh; Frederick William Kidd, M.D., Professor of Obstetrics and Gynæcology Royal College of Surgeons of Ireland; and W. J. Sinclair, M.D., Professor of Obstetrics and Gynæcology, Owen's College, Manchester. With these are associated as an editorial committee nine good men, with Dr. Eden as secretary, and after their names come those of quite a multitude of collaborators. If the "Journal" can escape the proverbial effects of having too many cooks the broth served up for its readers ought to be good, and, so far as we have tasted it in this first number, it has an admirable flavour. Dr. Cullingworth gives an analysis of 100 cases of uterine fibro-myomata in which the condition of the tumours and the complications to which they had given rise were verified by operation. He points out that at the present moment questions of surgical technique are of less importance than the larger question of the indications for operating. By some we are told that these tumours are for the most part absolutely harmless, and that their removal is necessary and justifiable only in certain rare instances in which they are producing symptoms dangerous to life. By others we are told that it is our duty to unlearn all that we have been taught as to the innocence of uterine fibro-myomata, as to their tendency to undergo spontaneous cure after the menopause, and as to their being very rarely fatal, and it is urged upon us that "the best treatment of fibroid tumours in general is their early removal." For the disentanglement of this problem what is evidently wanted is a more accurate knowledge than we yet possess of the life-history of these tumours, a knowledge based on a fairly long series of operations in which the tumours have been actually seen, handled, and laid open, and it is an analysis of such a series of operations that Dr. Cullingworth here gives. But, after all, important as is this contribution towards the solution of the problem, Dr. Cullingworth holds back from answering the question when to operate, and declines as yet to formulate the conclusions which are to be deduced from the facts set forth.

Dr. Peter Horrocks deals with the subject of contraction and retraction of muscular fibres, with special reference to the uterus. A good deal of the paper deals with physiological questions, and Dr. Horrocks takes much trouble to satisfy himself that when a muscle ceases to contract it relaxes and becomes soft but does not lengthen again except by the application of some force other than its own. From this he comes to this conclusion, among others, namely, that when on the expulsion of the fœtus the uterus contracts, it is not necessary for the prevention of hæmorrhage that *active* contraction should continue. Having contracted, until something dilates it, it will remain so, and "the sum total of the tone of the fibres of the retracted (relaxed) uterus is enough to secure the bleeding vessels." The active contraction of the empty uterus, when it is hard and easily felt, like a cricket ball, is only one phase, which is followed soon by the soft, less spheroidal, and impalpable phase, when the fibres are relaxed although possessing tone—tone enough to prevent hæmorrhage. During this perfectly natural phase of retraction great efforts are sometimes made by accoucheurs to alter this natural condition to the active state of contraction by kneading and rubbing the uterus, even when there is no excessive hæmorrhage going on and the pulse is good and normal in frequency. But to make the uterus feel "hard like a cricket-ball *all the time*" would be to set up a tetanic contraction which "would be abnormal, probably continuously painful, and might be accompanied by excessive hæmorrhage." Hence the teaching, "after the labour is over, the child and placenta born, it is quite unnecessary to trouble about the uterus if everything is satisfactory." Dr. Berry Hart makes "an attempt at an appreciation" of the position of obstetrics at the beginning of the twentieth century; Dr. W. J. Sinclair relates a case of tubal pregnancy of six months' development without rupture of the tube, and Dr. Murdoch Cameron one of fœtation in a rudimentary horn of a bicornute uterus. Then we have a critical essay by Dr. Arnold W. W. Lea on subarachnoid injection of cocaine, and after that a review of literature and reports of societies. Altogether a very good beginning for a new monthly journal on a special subject.

On Liquid Air and Its Application. By John Frick. (London: Furnival Press. 1901.)

This little pamphlet is intended to show to what useful purposes liquid air may be put, and there is much reason in some of the things the author says. Just as we warm our houses with fires in winter, so liquid air will cool them in summer. Taken into the furthest galleries of mines, liquid air will renew the atmosphere and solve the difficulties of mine ventilation. By the production of intense cold metallurgical operations at present practically unknown may be rendered possible; for example, steel may easily be crushed into powder. Again, by the production of cold all forms of food may be preserved, and our fur coats may be kept in good condition through the hottest summer. "Life is irksome in hot climates; liquid air will make Aden a pleasant place to live in." Liquid air again will enable us to dive to the bottom of deep water and will bring us back as readily to the surface, while "voyagers carrying with them but one inch of liquid air will be secure from drowning." It will also, we are told, be of great value in the abattoir. "In future cattle will not be bled to death; blood, the most nourishing part of the meat, will no longer flow into the blood-gutter, but the stunned animal will be frozen to death, and all the nutriment juices of the meat kept coagulated until retailed by the butcher," which, be it said, does not appear to us to be a particularly delightful notion. On the other hand we quite agree that theatres and crowded halls urgently need liquid air, in fact air of any sort. These are but a few of the hundreds of uses to which we may put liquid air when once it becomes an article of commerce. This is where the difficulty arises, and it is somewhat disheartening to find the author say that the various types of machine which he has studied are "impracticable for the commercial production of liquid air." Still there is hope, for the author describes an apparatus which he has invented for the purpose, by which he is able to "ensure the production of any quantity of liquid air easily and cheaply." That liquid air if it could be cheaply made, retailed, and distributed, would be a very useful commodity no one will deny, but if we may be allowed to alter the spelling of an old adage of the cookery books, we would say "first catch your air."

